# Combining CHA_2_DS_2_-VASc score into RCRI for prediction perioperative cardiovascular outcomes in patients undergoing non-cardiac surgery: a retrospective pilot study

**DOI:** 10.1186/s12871-021-01496-2

**Published:** 2021-11-09

**Authors:** Song-Yun Chu, Pei-Wen Li, Fang-Fang Fan, Xiao-Ning Han, Lin Liu, Jie Wang, Jing Zhao, Xiao-Jin Ye, Wen-Hui Ding

**Affiliations:** 1 Department of Cardiology, Peking University First Hospital, No. 8, Xishiku Street, Xicheng District, Beijing, 100034 People’s Republic of China; 2 Department of Cardiology, Drum Tower Hospital, Nanjing University Medical School, Nanjing, China

**Keywords:** Perioperative cardiovascular outcome, Non-cardiac surgery, CHA_2_DS_2_-VASc score, RCRI

## Abstract

**Background:**

Treatment decisions in patients undergoing non-cardiac surgery are based on clinical assessment. The Revised Cardiac Risk Index (RCRI) is pragmatic and widely used but has only moderate discrimination. We aimed to test the efficacy of the CHA_2_DS_2_-VASc score and the combination of CHA_2_DS_2_-VASc and RCRI to predict perioperative risks for non-cardiac surgery.

**Methods:**

This pre-specified analysis was performed in a retrospective cohort undergoing intra-abdominal surgery in our center from July 1st, 2007 to June 30th, 2008. The possible association between the baseline characteristics (as defined by CHA_2_DS_2_-VASc and RCRI) and the primary outcome of composite perioperative cardiac complications (myocardial infarction, cardiac ischemia, heart failure, arrhythmia, stroke, and/or death) and secondary outcomes of individual endpoints were explored using multivariate Logistic regression. The area under the receiver operating characteristic curve (*C*-statistic) was used for RCRI, CHA_2_DS_2_-VASc, and the combined models, and the net reclassification improvement (NRI) was calculated to assess the additional discriminative ability.

**Results:**

Of the 1079 patients (age 57.5 ± 17.0 years), 460 (42.6%) were women. A total of 83 patients (7.7%) reached the primary endpoint. Secondary outcomes included 52 cardiac ischemic events, 40 myocardial infarction, 20 atrial fibrillation, 18 heart failure, four strokes, and 30 deaths. The endpoint events increased with the RCRI and CHA_2_DS_2_-VASc grade elevated (*P* < 0.05 for trend). The RCRI showed a moderate predictive ability with a C-statistics of 0.668 (95%CI 0.610–0.725) for the composite cardiac outcome. The C-statistics for the CHA_2_DS_2_-VASc was 0.765 (95% CI 0.709–0.820), indicating better performance than the RCRI (*p* = 0.011). Adding the CHA_2_DS_2_-VASc to the RCRI further increased the C-statistic to 0.774(95%CI 0.719–0.829), improved sensitivity, negative predictive value, and enhanced reclassification in reference to RCRI. Similar performance of the combined scores was demonstrated in the analysis of individual secondary endpoints. The best cut-off of a total of 4 scores was suggested for the combined CHA_2_DS_2_-VASc and RCRI in the prediction of the perioperative cardiac outcomes.

**Conclusions:**

The CHA_2_DS_2_-VASc score significantly enhanced risk assessment for the composite perioperative cardiovascular outcome in comparison to traditional RCRI risk stratification. Incorporation of CHA_2_DS_2_-VASc scores into clinical-decision making to improve perioperative management in patients undergoing non-cardiac surgery warrants consideration.

**Supplementary Information:**

The online version contains supplementary material available at 10.1186/s12871-021-01496-2.

## Introduction

Current practice guidelines [1] recommend risk stratification with the validated tools to predict the risk of perioperative major adverse cardiac events in patients undergoing non-cardiac surgery.

The Revised Cardiac Risk Index (RCRI), American College of Surgeons National Surgical Quality Improvement Program (NSQIP) Myocardial Infarction and Cardiac Arrest (MICA), and American College of Surgeons NSQIP Surgical Risk Calculator are the risk assessment tools listed in the guidelines. The latter two newer tools have been created by the American College of Surgeons, in which more attention has been paid to the specific type and location of surgeries and functional status of patients. Although the detailed data collection might provide more precise risk prediction, the need for trained nurses and time-consuming web-based or spreadsheet for calculation are obstacles in daily practice. Other limitations include the difficulties in the evaluation of the physical status and less validation in the external population. In contrast, the RCRI is a simple, validated, and well-accepted tool to assess perioperative cardiovascular complications. However, emerging clinical cases has highlighted limitations in the predictive value of the score. For example, age and gender differences have not been considered. And the weight of every predictor is assumed as the same.

The CHA_2_DS_2_-VASc (congestive heart failure, hypertension, age, diabetes, stroke, vascular disease, and female gender) score is a stroke risk stratification system in patients with non-valvular AF. Recently, the usage of the CHA_2_DS_2_-VASc score has been extended beyond the original scenario to predict other adverse cardiovascular outcomes such as heart failure, myocardial infarction, and death [2, 3]. Moreover, the efficacy of the CHA_2_DS_2_-VASc score remains even in subjects without AF [4].

We hypothesized that the addition of the CHA_2_DS_2_-VASc score to the RCRI would improve perioperative cardiovascular outcomes prediction in individuals undergoing non-cardiac surgery. We tested this hypothesis in a large cohort of patients receiving general surgery procedures based on a year-round registry.

## Methods

### Patients and data collection

The study was conducted at the surgical department in our tertiary hospital and was approved by the Ethics Committee on Clinical Investigation of our hospital. A waiver of informed consent from patients was obtained owing to the retrospective nature of the study. From July 1st, 2007 to June 30th, 2008, consecutive eligible adult patients (age ≥ 18) hospitalized for intra-abdominal surgery were included to establish the study cohort [5]. The medical records were retrieved to capture data on patients’ characteristics, including demographics, medical history, laboratory, imaging, and perioperative variables. The medical history variables were defined by the presence of eligible diagnosis codes [International Classification of Diseases, Tenth Revision (ICD-10)]. Intra-abdominal surgery was defined as open abdominal surgery (laparoscopic surgery was excluded) involving the stomach, intestine, bile bladder and duct, liver, spleen, duodenum, pancreas, colon and rectum.

The perioperative period was defined as the interval between admission and discharge. Perioperative cardiovascular events were specified defined as (1): perioperative myocardial infarction (MI): detection of a rise and/or fall of cardiac biomarker [cardiac troponin I (cTnI)] value with at least one value above the 99th percentile upper reference limit (URL) and with at least one of the following criterion including symptoms of ischemia, new ST-T changes or left bundle branch block (LBBB), development of pathological Q waves in the ECG, imaging evidence of new loss of viable myocardium, and identification of an intracoronary thrombus by angiography, according to the Fourth Universal Definition of Myocardial Infarction [6] (2); cardiac ischemic events: including MI, angina, transient or prolonged ST-segment change comparing to baseline ECG (3); acute heart failure: at least one of the following criteria was met: exertional, resting, and/or paroxysmal nocturnal dyspnea, new onset bilateral rales, S3 heart sound, fluid overload/peripheral edema that need diuretics, signs of heart failure on chest X-ray or echocardiography [7–9] (4); arrhythmia: new-onset arrhythmia recorded by ECG during index hospitalization, including ventricular fibrillation/tachycardia, atrial fibrillation, atrial flutter, supraventricular tachycardia, II or III degree atrioventricular block, and long R-R interval (> 2 s) (5); stroke: a focal neurological deficit lasting 24 h or until death or if the deficit lasted < 24 h and there was a clinically relevant lesion on brain imaging (6); all-cause death; and (7) composite cardiovascular events: occurrence of at least one of the events mentioned above.

The primary endpoint was defined as the occurrence of the composite cardiovascular events (MI, ischemic events, acute heart failure, arrhythmia, stroke, and/or death). The secondary endpoint was defined as the occurrence of the aforementioned pre-specified perioperative cardiac events.

### Statistical analyses

Continuous data were described as mean ± SD or median and interquartile range (IQR), and categorical data were expressed as numbers and percentages.

Revised Cardiac Risk Index (RCRI): renal insufficiency (creatinine≥2 mg/dL), insulin-dependent diabetes mellitus, heart failure, ischemic heart disease, cerebrovascular accident or TIA, intra-thoracic, intra-abdominal, or supra-inguinal vascular surgery; each one calculated as one score.

CHA_2_DS_2_-VASc includes a history of congestive heart failure (1 point), hypertension (1 point), age 65–74 (1 point) or ≥ 75 years (2 points), diabetes mellitus (1 point), stroke, transient ischemic attack, or thromboembolism (2 points), vascular disease (1 point), and sex category (female).

The association of baseline variables included in the RCRI and CHA_2_DS_2_-VASc score system and perioperative cardiovascular events were analyzed with univariate Logistic regression. Multivariate Logistic regression was then built with the confounders included if their univariate significance was less than 0.1. Odds ratios (OR) of the risks of the perioperative cardiovascular events were given with 95% confidence intervals (CI).

We constructed models for the association of the scoring grade (0, 1, 2, and ≥ 3) with incident perioperative cardiovascular outcomes. Model 1, 2, and 3 was the model using RCRI, CHA_2_DS_2_-VASc, and the combination, respectively. The prognostic accuracy of the models was compared using the area under the curve derived from receiver operating characteristic (ROC) curves (C-statistic). Sensitivity, specificity, positive predictive, and negative predictive values were reported. We used the Hosmer-Lemeshow χ^2^ statistic to evaluate model calibration. The ability of the CHA_2_DS_2_-Vasc score to enhance discrimination and correctly reclassify patients were additionally tested with the categorical net reclassification improvement (NRI) using model 1 as the benchmark for comparison. Reclassification categories were defined as < 2, 2 to 3%, and > 3% of perioperative myocardial infarction, < 2%, 2–5, > 5% of cardiac ischemic events, < 1, 1 to 2%, and > 2% of heart failure, stroke, and all-cause death, < 3%, 3–6, and > 6% of total perioperative cardiovascular events [9–11].

Analyses were performed using the Statistical Package for the Social Sciences (SPSS) version 22.0 (SPSS, Inc., Chicago, IL, USA) and R (version 3.4.3, http://www.R-project.org). A *p* < 0.05 (2-tailed) was considered statistically significant.

## Results

### Baseline characteristics of the study cohort

A total of 1258 consecutive subjects who underwent intra-abdominal surgery between July 1st, 2007 and June 30, 2008 were evaluated. Of these, 179 patients with missing baseline information or repeated surgery in the same period were excluded. A total of 1079 patients (age 57.5 ± 17.0 years, 460 women) were included in the analysis. Overall, this cohort had considerable comorbidities, including hypertension, diabetes, cardiovascular and cerebrovascular disease. Most of the surgeries (74.2%) were elective and complicated surgeries (involving more than three major organs) constituted less than 10% of the procedures (Table 1).

**Table 1:** Characteristics of patients undergoing intra-abdominal surgery

Characteristics	Total population (***N*** = 1079)
Age (yrs.), Mean ± SD	57.5 ± 17.0
Women, n (%)	460 (42.6)
**Medical history**	
Hypertension, n (%)	313 (29.0)
Diabetes, n (%)	154(14.3)
Insulin-dependent diabetes, n (%)	42 (3.9)
Coronary heart disease, n (%)	105 (9.7)
Congestive heart failure, n (%)	15 (1.4)
Cerebrovascular disease, n (%)	57 (5.3)
Ischemic stroke, n (%)	52 (4.8)
Chronic kidney disease, n (%)	78 (7.2)
Creatinine > 2 mg/dL, n(%)	16(1.5)
Vascular disease, n (%)	111 (10.3)
**Surgery and anesthesia**	
Emergent surgery, n (%)	278 (25.8)
Complicated surgery, n (%)	98 (9.1)
General anesthesia, n (%)	358 (33.2)
Epidural, spinal, combined spinal and epidural anesthesia, n(%)	721(66.8)
Surgery time (h) (median, IQR)	2.8 (1.7, 4.3)
Blood loss (mL) (median, IQR)	100 (0, 300)

SD, standard deviation; IQR, interquartile range

### Incidences of the perioperative cardiovascular events

A total of 83 patients (7.7%) reached the primary outcome. Secondary outcomes included 52 cases of cardiac ischemic events, 40 cases of perioperative MI, 24 cases of arrhythmia (20 were atrial fibrillation), 18 cases of acute heart failure, 4 cases of ischemic stroke, and 30 cases of all-cause deaths.

### RCRI, CHA_2_DS_2_-VASc, and the combination to predict perioperative cardiac outcomes

The Logistic regression models were used to estimate the association between the characteristics identified by CHA_2_DS_2_-VASc or RCRI and perioperative cardiovascular outcomes in the cohort.

**Fig. 1:**
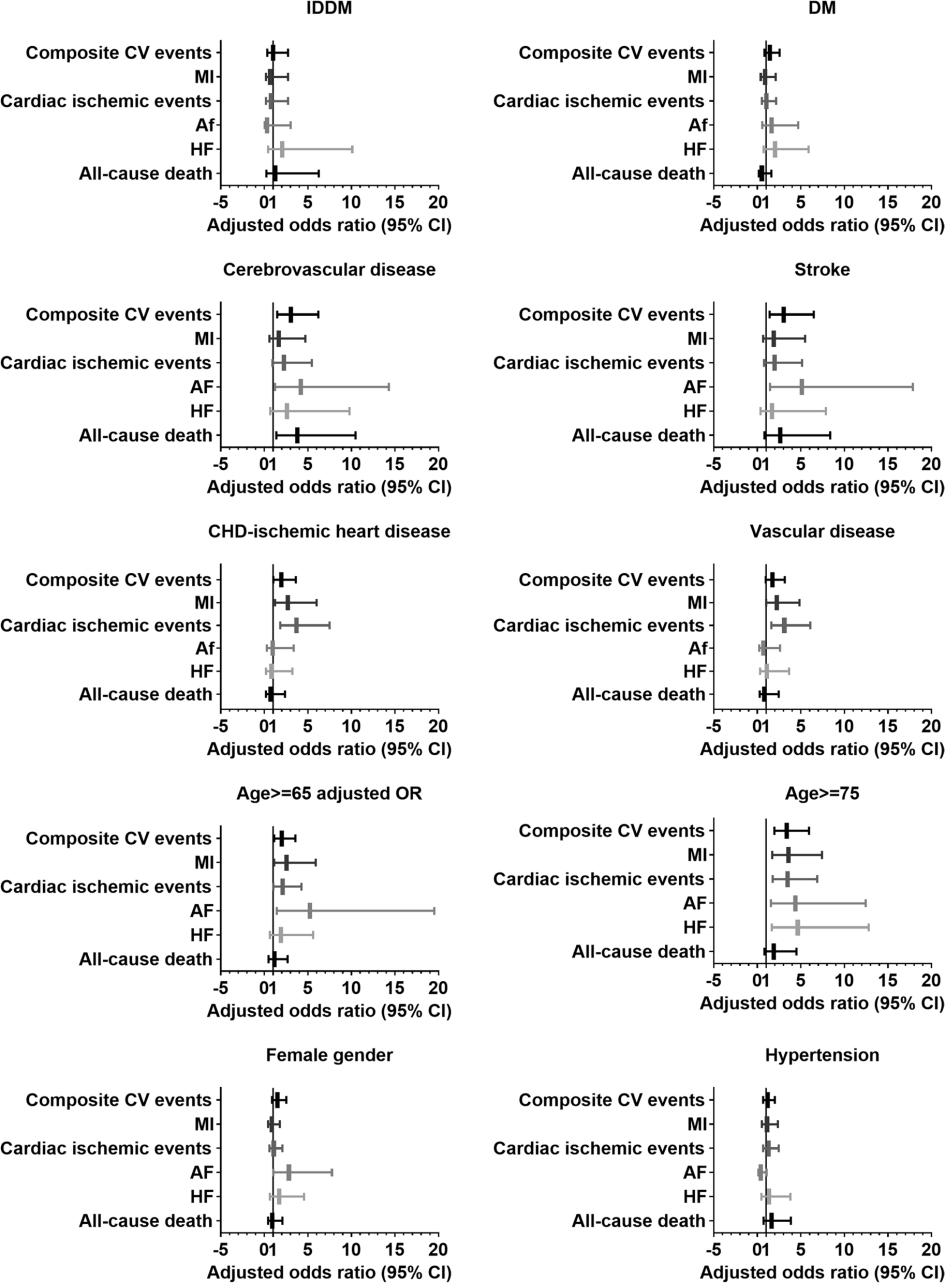
Association of the clinical characteristics modified or introduced by CHA_2_DS_2_–VASc with the primary and secondary perioperative cardiovascular outcomes. Odds ratios were calculated using multivariate Logistic regression. Abbreviations: MI: myocardial infarction; HF: heart failure; AF: atrial fibrillation; DM: diabetes; IDDM: insulin-dependent diabetes; TIA: transient ischemic attack; CV: cardiovascular; CI: confidence interval

The elements of the two scoring systems both identified elevated risks of the primary and secondary endpoint events. History of heart failure, diabetes, and cerebrovascular disease were predictors shared by the two systems. Due to the different definitions of diabetes and cerebrovascular disease in RCRI and CHA_2_DS_2_-VASc, subtle different risks for cardiac outcomes were suggested. As the most common vascular disease was coronary artery disease, the ischemic heart disease in RCRI and vascular disease in CHA_2_DS_2_-VASc associated with similar risks for specific cardiac endpoints. Among the new factors introduced by CHA_2_DS_2_-VASc, advanced age was a robust predictor for most perioperative cardiac events. Age more than 75 years old remained as independent risk factors for all the major adverse cardiac events except for death. Gender and hypertension were weaker predictors (Fig. 1, Supplementary Table 1).

**Fig. 2:**
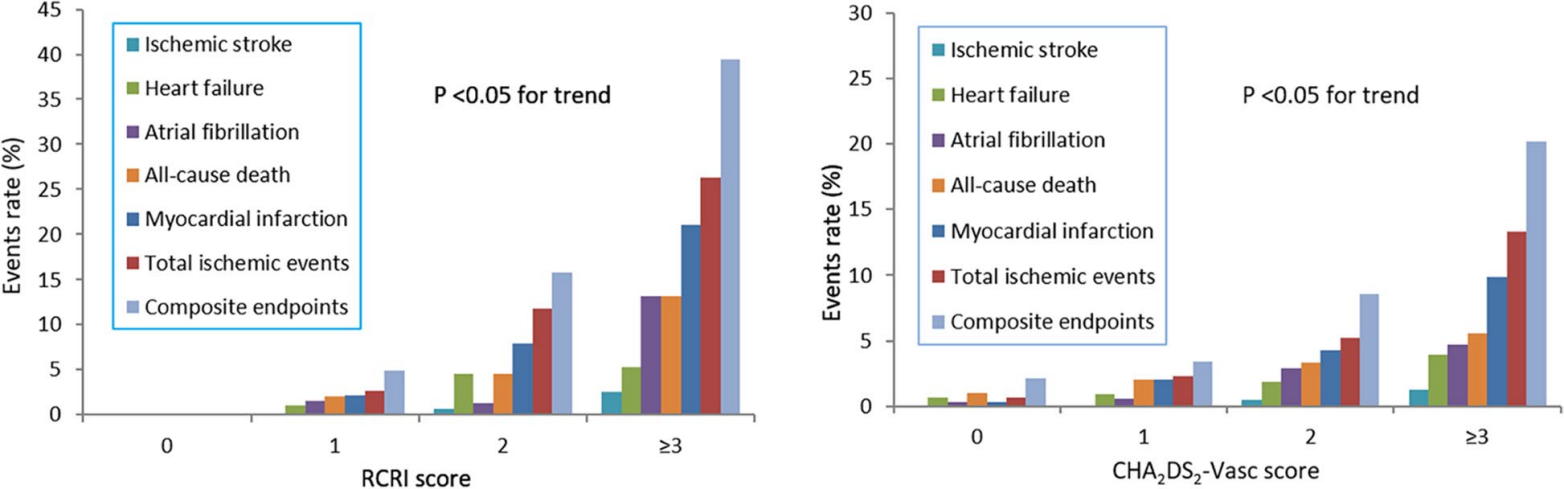
The incidence of perioperative cardiovascular events stratified by RCRI grade and CHA_2_DS_2_–VASc score grade. The incidence of perioperative cardiovascular endpoints increased in a significantly graded fashion with both the established RCRI and CHA_2_DS_2_–VASc score

The primary endpoint of composite perioperative cardiac events, as well as all the secondary outcomes of MI, ischemic events, atrial fibrillation, heart failure, stroke, and death increased progressively, both with the RCRI and CHA_2_DS_2_-VASc grade elevated (*P* < 0.05 for trend). For comparison, a steeper association in relative risk increase of cardiac endpoints was observed with the RCRI increased (Fig. 2, Supplementary Table 2 and 3).

In the analysis of perioperative risk prediction models, the calibration, discrimination, and risk reclassification were shown in Table 2.

**Table 2:** Assessment of the RCRI, CHA_2_DS_2_-VASc and the combination as predictor for perioperative cardiovascular events

Variable	C-statistic (95% CI)	Sensitivity	Specificity	X^**2**^ (***P*** value) ^**b**^	PPV	NPV	NRI (95% CI)
**Composite endpoints**							
RCRI	0.668(0.610–0.725)	0.470	0.848	0.0001(1.000)	0.76	0.62	1 [Reference]
CHA_2_DS_2_-VASc	0.765(0.709–0.820), P^a^ = 0.011*	0.783	0.621	4.996(0.758)	0.67	0.74	0.308(0.172–0.445), P^a^ < 0.001*
CHA_2_DS_2_-VASc +RCRI	0.774(0.719–0.829), P^a^ < 0.001*	0.542	0.879	4.315(0.828)	0.82	0.66	0.308(0.172–0.445), P^a^ < 0.001*
**Myocardial infarction**							
RCRI	0.678(0.595–0.760)	0.500	0.836	0.025(1.000)	0.75	0.63	1 [Reference]
CHA_2_DS_2_-VASc	0.775(0.706–0.844), P^a^ = 0.028*	0.800	0.604	7.563(0.477)	0.67	0.75	0.506(0.275–0.737), P^a^ < 0.001*
CHA_2_DS_2_-VASc +RCRI	0.791(0.725–0.857), P^a^ = 0.003*	0.750	0.674	5.329(0.722)	0.70	0.73	0.501(0.313–0.688), P^a^ < 0.001*
**Cardiac ischemic events**							
RCRI	0.698(0.627–0.770)	0.539	0.842	0.261(1.000)	0.77	0.65	1 [Reference]
CHA_2_DS_2_-VASc	0.779(0.716–0.841), P^a^ = 0.030*	0.808	0.610	5.833 (0.666)	0.67	0.76	0.278(0.123–0.433), P^a^ < 0.001*
CHA_2_DS_2_-VASc +RCRI	0.792(0.732–0.853), P^a^ = 0.002*	0.673	0.772	3.604(0.891)	0.75	0.70	0.324(0.192–0.455), P^a^ < 0.001*
**Atrial fibrillation**							
RCRI	0.617(0.501–0.732)	0.250	0.972	1.203(0.997)	0.90	0.56	1 [Reference]
CHA_2_DS_2_-VASc	0.802(0.701–0.902), P^a^ = 0.012*	0.800	0.701	6.260(0.618)	0.73	0.78	0.593 (0.297–0.889), p^a^ < 0.001*
CHA_2_DS_2_-VASc +RCRI	0.833(0.739–0.927), P^a^ < 0.001*	0.800	0.752	3.882(0.868)	0.76	0.79	0.593 (0.297–0.889), P^a^ < 0.001*
**Heart failure**							
RCRI	0.668(0.548–0.789)	0.500	0.832	0.436(0.999)	0.75	0.62	1 [Reference]
CHA_2_DS_2_-VASc	0.727(0.593–0.861), p^a^ = 0.411	0.667	0.698	1.452(0.994)	0.69	0.68	0.315 (−0.016–0.646), P^a^ = 0.062
CHA_2_DS_2_-VASc +RCRI	0.743(0.623–0.863), P^a^ = 0.188	0.722	0.655	0.429(1.000)	0.68	0.70	0.315(−0.016–0.646), p^a^ = 0.062
**Stroke**							
RCRI	0.678(0.379–0.978)	0.500	0.827	0.019(1.000)	0.74	0.62	1 [Reference]
CHA_2_DS_2_-VASc	0.924(0.832–1.000), P^a^ = 0.039*	1.000	0.694	1.848(0.985)	0.77	1.00	0.431 (−0.059–0.922), p^a^ = 0.085
CHA_2_DS_2_-VASc +RCRI	0.952(0.894–1.000), P^a^ = 0.048*	1.000	0.786	2.876(0.942)	0.82	1.00	0.487(−0.003–0.977), P^a^ = 0.052
**All-cause death**							
RCRI	0.623(0.530–0.717)	0.400	0.830	0.005(1.000)	0.70	0.58	1 [Reference]
CHA_2_DS_2_-VASc	0.676(0.578–0.775), P^a^ = 0.076	0.667	0.626	1.702(0.989)	0.64	0.65	0.414(0.365–0.463), P^a^ < 0.001*
CHA_2_DS_2_-VASc +RCRI	0.719(0.621–0.817), P^a^ = 0.015*	0.433	0.884	0.427(1.000)	0.79	0.61	0.390(0.344–0.436), P^a^ < 0.001*

RCRI: Revised Cardiac Risk Index, includes renal insufficiency (creatinine≥2 mg/dL), insulin-dependent diabetes mellitus, heart failure, ischemic heart disease, cerebrovascular accident or TIA, intra-thoracic, intra-abdominal, or supra-inguinal vascular surgery; each one calculated as 1 score

CHA_2_DS_2_-VASc includes history of congestive heart failure (1 point), hypertension (1 point), age 65–74 (1 point) or ≥ 75 years (2 points), diabetes mellitus (1 point), stroke, transient ischemic attack or thromboembolism (2 points), vascular disease (1 point), and sex category (female)

a. Compared with RCRI grade alone; b. Hosmer-Lemeshow χ^2^ statistic. PPV, positive predictive value; NPV, negative predictive value; NRI, net reclassification improvement; CI, confidence interval. * p < 0.05

**Fig. 3:**
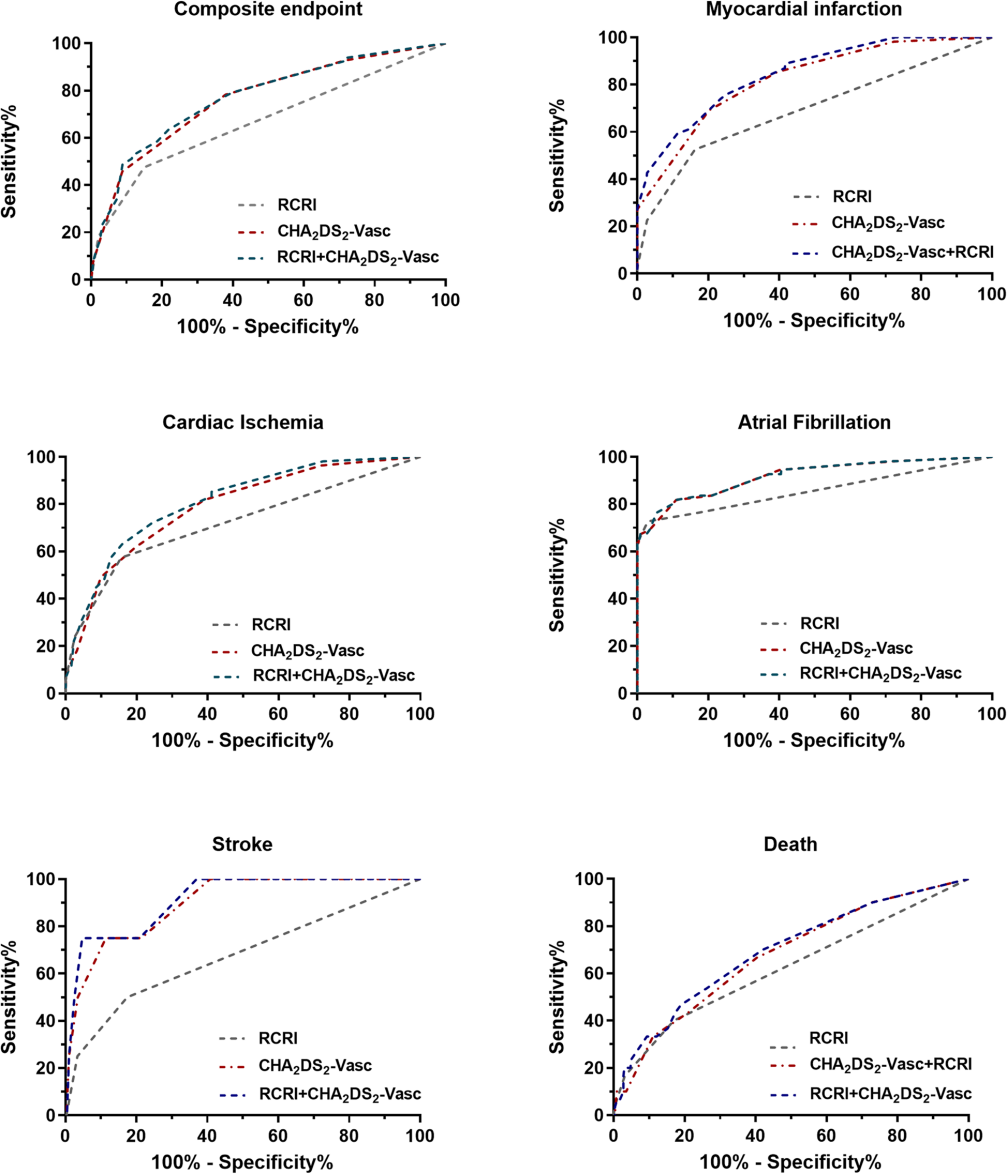
The ROC curves for RCRI, CHA_2_DS_2_–VASc, and the combined in predicting the primary and secondary perioperative cardiac endpoints

For the primary endpoint of the composite cardiac events, the Hosmer-Lemeshow test for goodness-of-fit of the models indicated good calibration for the RCRI, CHA_2_DS_2_-VASc score, and the combination (all *P* > 0.05). The RCRI showed a moderate predictive ability with a *C*-statistics of 0.668 [95% confidence interval, (CI) 0.610–0.725]. The *C*-statistics for the CHA_2_DS_2_-VASc was 0.765 (95% CI 0.709–0.820), indicating better performance than the RCRI (*p* = 0.011). Adding the CHA_2_DS_2_-VASc to the RCRI further increased the *C*-statistic to 0.774(95%CI 0.719–0.829, *p* < 0.001). The CHA_2_DS_2_-VASc and the combined two scoring systems also improved the sensitivity and negative predictive value. Further, the CHA_2_DS_2_-VASc alone and the combination of the CHA_2_DS_2_-VASc and RCRI also significantly enhanced reclassification for perioperative cardiac events in reference to RCRI alone. (Table 2, Fig. 3).

For secondary endpoints of individual perioperative cardiovascular events, the RCRI showed a moderate predictive ability (*C*-statistics 0.617–0.698). The *C*-statistics for the CHA_2_DS_2_-VASc ranged between 0.676 and 0.924, depending on the specific postoperative outcome examined. In general, the CHA_2_DS_2_-VASc performed at least as well as the RCRI in most perioperative cardiac events with higher *C*-statistics. Specifically, the CHA_2_DS_2_-VASc significantly improved prediction for myocardial infarction, cardiac ischemic events, atrial fibrillation, and stroke when compared with the RCRI [C-statistic (95% CI): 0.775(0.706–0.844) vs. 0.678(0.595–0.760), *P* = 0.028; 0.779(0.716–0.841) vs. 0.698(0.627–0.770), *p* = 0.030; 0.802(0.701–0.902) vs. 0.617(0.501–0.732), *p* = 0.012; and 0.924(0.832–1.000) vs. 0.678(0.379–0.978), *p* = 0.039; respectively). Adding the CHA_2_DS_2_-VASc to the RCRI further increased the *C*-statistic (95%CI) significantly for perioperative MI, cardiac ischemic events, atrial fibrillation, stroke, and all-cause death [C-statistic (95% CI): 0.791(0.725–0.857) vs. 0.678(0.595–0.760), *p* = 0.003; 0.792(0.732–0.853) vs. 0.698(0.627–0.770), *p* = 0.002; 0.833(0.739–0.927) vs. 0.617(0.501–0.732), *P* < 0.001; 0.952(0.894–1.000) vs. 0.678(0.379–0.978), *p* = 0.048; 0.719(0.621–0.817) vs. 0.623(0.530–0.717), *p* = 0.015, respectively]. The CHA_2_DS_2_-VASc suggested a small to moderate degree of reclassification for MI, ischemic events, death, and atrial fibrillation (NRI 0.278–0.593). The combination of the CHA_2_DS_2_-VASc score and RCRI showed comparable or even higher degree of reclassification than CHA_2_DS_2_-VASc score. (Table 2, Fig. 3).

### The best cut-off for the combined RCRI and CHA_2_DS_2_-VASc scores in the prediction of the composite perioperative cardiovascular events

Optimal cut-off values were extracted from ROC curve analyses of RCRI, CHA_2_DS_2_-VASc, and the combined scores. Specifically, a cut-off of 1.5 suggested elevated composite perioperative cardiac risks by RCRI, CHA_2_DS_2_-VASc, and the combination of the two scoring systems suggested a sum score of 3.5 predicted elevated composite perioperative cardiac events. Considering the cut-off of 2 scores were suggested originally both by RCRI and CHA_2_DS_2_-Vasc system for elevated risks, a total score of 4 also provided the best trade-off between sensitivity (62.7%) and specificity (78.2%) for the convenience of the clinical usage (Table 3).

**Table 3:** Estimate the best cut-off of RCRI, CHA_2_DS_2_-VASc Score and the combined scores in predicting the composite perioperative cardiac events

Score	C-statistic (95% CI)	Best cutoff	Sensitivity	Specificity	Number for diagnose
**RCRI**	0.667(0.610–0.724)	1.5	0.470	0.848	3.1
**CHA**_**2**_**DS**_**2**_**-VASc**	0.764(0.709–0.819)	1.5	0.783	0.621	2.5
**CHA**_**2**_**DS**_**2**_**-VASc + RCRI**	0.772(0.718–0.826)	3.5	0.627	0.782	2.4

## Discussion

The main finding of this study is that risk stratification for perioperative cardiovascular outcomes can be improved by combining CHA_2_DS_2_-VASc score with RCRI. RCRI has high specificity to predict adverse cardiac prognosis; its integration with CHA_2_DS_2_-VASc improves sensitivity and the discriminating ability for cardiovascular endpoints.

Cardiovascular complications constituted the leading cause of adverse perioperative outcomes. Current guidelines recommend risk stratification for patients receiving non-cardiac surgery. Risk stratification provides the guide to physicians with respect to the important decision as specific detailed evaluation and precautions are needed. The RCRI is a simple and widely used scheme. However, it has been reported that the RCRI has only moderate discrimination [12]. While RCRI equal to or more than 2 calls on an alert, greater uncertainty arises for those with a score of 0 to 1. For example, the subjects with an RCRI of 1 were preponderant in our cohort, accounting for 82.5%. At the same time, about half of the endpoint events occurred in these populations, although their RCRI identified them as low risk.

Any attempt for additional improvement of risk stratification is of clinical interest. Some prior researchers have challenged the limitations of the RCRI and explored adjusting the factors either by removing or adding on new parameters in the scoring systems to improve the final risk stratification or the discrimination of the index [13, 14]. Newer stratification system such as NSQIP Surgical Risk Calculator has also been developed to solve the clinical needs. However, the more complex index might achieve greater accuracy but at the expense of ease of use. The hope to derive a simple and ready-to-use tool in routine practice is always attractive. We intend to introduce another validated scoring scheme that might complement the pre-existent index.

The CHA_2_DS_2_-VASc score is a stroke risk stratification system in patients with non-valvular AF. Recently, the usage of the CHA_2_DS_2_-VASc score has been explored in other scenarios [2, 3]. Moreover, the efficacy of the CHA_2_DS_2_-VASc score in predicting cardiac outcomes remains even in subjects without AF [4]. The primary values of CHA_2_DS_2_-VASc are their easy use and ability to identify low-risk patients who do not require anticoagulation [15].

In the present study, we attempted to combine the two scoring systems to evaluate perioperative cardiac hazard. Both scoring systems emphasize traditional atherosclerotic risk factors, including the history of diabetes, coronary heart disease, cerebrovascular disease/ischemic stroke, and congestive heart failure. Each of these characteristics has been demonstrated as a potent predictor for adverse cardiovascular prognosis, although the subtle difference in definition and weight of them have been adopted in the two scoring systems. Indeed, in our Logistic regression model, these medical conditions correlated with increased risks for cardiac complications. As in prior research [14], though, we have found that different factors in the model might not be equal in predictive efficacy. For instance, insulin-dependent diabetes seemed to be the poorest predictor. As in the original description, the removal of it does not affect the final risk stratification or the discrimination of the index [9]. When diabetic history was used as an alternative in the CHA_2_DS_2_-VASc scoring system, it correlated with elevated cardiac risks, only not as an independent predictor in the multivariate analysis. In contrast, preoperative serum creatinine ≥2.0 mg/dL strongly indicated adverse cardiac outcomes. The history of cerebrovascular disease was a predictor of multiple cardiac complications. Notably, the ischemic stroke history correlated with a dramatically elevated perioperative stroke risk, although the scarcity of the stroke events precluded further multivariate analysis. And the perioperative stroke rate of 1.3% for patients with CHA2DS2-VASc > = 3 was in line with the early reported incidence of perioperative stroke in non-cardiac, non-neurologic, and non-major vascular surgery that ranged from approximately 0.1 to 1.9% depending on associated risk factors [16–18]. This highlighted the importance of stroke history, coincident with the more weight of it being assigned in the CHA_2_DS_2_-VASc scheme.

An important predictor introduced by the CHA_2_DS_2_-VASc score is age. Although no correlation with complications was found in the original RCRI derivative population, age has been taken as one of the variables in several risk stratification systems [19, 20]. And with the life expectancy of the general population increased, it is estimated that the elderly require surgery almost four times more often than younger ones [21]. Except for bearing more atherosclerotic risks, the elderly have reduced compliance of cardiovascular beds and thus decreased functional reserve, which predisposes them to cardiac dysfunction [22]. On the other hand, insults of perioperative stresses might induce coronary lesion being unstable and cardiac acute decompensation [23]. In our study, age ≥ 65 and ≥ 75 years were both predictors for perioperative cardiac complications and mostly acted as independent risk factors. Age ≥ 75 years was linked to even higher risk as it does in the original CHA_2_DS_2_-VASc stratification. On the contrary, gender differences seemed to be unrelated to any cardiac outcomes. In prior research for the same population [5], however, female gender was an independent risk factor for perioperative heart failure in patients with age ≥ 65. We retained the gender information in the novel index in hopes that each clinical risk factor might enhance prognostic accuracy.

Hypertension has been established as a powerful predictor of cardiovascular morbidity and mortality. The prevalence of hypertension makes it one of the most common comorbidities seen in the perioperative setting, and that uncontrolled hypertension is suggested to be the most common medical reason for surgery cancellation [24]. However, outcomes of hypertension have not been adequately studied in the perioperative setting. And a J-shaped curve of optimal perioperative diastolic blood pressure management has been suggested [25]. The widely used RCRI does not include systemic hypertension as a risk factor. On the other hand, the fact that hypertension is linked to perioperative acute cardiogenic pulmonary edema suggested the link through exacerbated diastolic dysfunction [26, 27]. In our cohort, patients with hypertension accounted for one-third of the population. And hypertension has been recognized as a risk factor for most cardiac endpoints. Therefore, adding on hypertension could be complimentary to the RCRI model.

The RCRI showed a moderate predictive ability for all the perioperative cardiovascular endpoints. CHA_2_DS_2_-VASc alone and combining the CHA_2_DS_2_-VASc and RCRI increased the discriminating capacity for perioperative MI, ischemic events, atrial fibrillation, stroke, and composite events. We believe that the complementary factors offered by CHA_2_DS_2_-VASc led to the improvement of the performance of the newer risk stratification tools. These were also demonstrated by the significantly enhanced reclassification and increased negative predictive value. Thus, patients with RCRIs that qualify them as lower risk might need additional assessment, while the patients identified by the newer models as low risk might precede surgery safely. More accurate risk assessment allows us to use tailored therapy in patients who will achieve benefit while avoid exposing other patients to unnecessary risk. Therefore, incorporating the CHA_2_DS_2_-VASc risk score into decision-making in patients undergoing surgery may bring us closer to our goal of precision medicine.

### Limitations

The analysis was performed in a modest-sample-sized specific population undergoing intra-abdominal surgery in our center. Because all the patients received intra-abdominal operation as defined in RCRI as one of the high-risk surgery types, patients with an RCRI of 0 were not included in our cohort. Therefore, we do not have data regarding the performance of the combined risk score in patients at the lowest risk of perioperative cardiovascular outcomes as defined by the RCRI. However, our results suggest the potential of the CHA_2_DS_2_-VASc risk score to identify patients categorized into this lower risk category who have a significantly elevated risk that may warrant further evaluation. And the hypothesis needs to be tested in patients across the full spectrum of RCRI risk scores. As for the study cohort, coronary heart disease as one of the RCRI predictors was less prevalent than the original derivation. And the seemingly paradoxical higher prevalence of MI in our cohort than that seen in the original derivation might be explained by the evolving use of high-sensitivity troponin measurements as the gold standard for diagnosing MI. However, it has been demonstrated any troponin release correlated to adverse cardiac outcomes. The CHA_2_DS_2_-VASc score was originally proposed by Gregory Lip for the estimation of stroke risk in patients with atrial fibrillation and the score has been used in different situations other than the original one. There is a risk for this usage represent the effect of collinearity other than a pathophysiological explanation. However, the score scheme has considered age, gender and multiple atherosclerotic risk factors and we have demonstrated the association between the clinical characters identified by the score and the patient’s perioperative cardiac outcomes in the multivariate regression.

Finally, the add-on of CHA_2_DS_2_-VASc risk score increased the sensitivity of the prediction model, which might have also result in over-diagnosis and overtreatment. The best cut-off analysis, though, has suggested at least total scores of 4 of the two score-systems indicating elevated risks for perioperative cardiovascular events, and the number for diagnosis was less than 3. Due to the hypothesis-generating nature of our combined risk score, it requires validation and refinement in additional studies that should include populations outside of the highly selected clinical cohort. Therefore, the present finding is the first step for incorporating of CHA_2_DS_2_-VASC in the perioperative evaluation, and prospective validation in different types of surgery is mandatory.

## Conclusions

A combined risk score was developed that significantly enhanced risk assessment for perioperative cardiovascular outcomes compared with traditional clinical risk stratification RCRI. Incorporating the CHA_2_DS_2_-VASc scores into RCRI to define preventative and therapeutic management in patients undergoing non-cardiac surgery warrants consideration.

## Supplementary Information

**Additional file 1.**

## Data Availability

The datasets used and/or analysed during the current study are available from the corresponding author on reasonable request.

## Abbreviations

AF: atrial fibrillation
CHA_2_DS_2_-Vasc: Congestive heart failure, Hypertension, Age, Diabetes, Stroke, Vascular disease, and female gender
CI: confidence interval
cTnI: cardiac troponin I
CV: cardiovascular
DM: diabetes
HF: heart failure
ICD-10: International Classification of Disease, the 10th revision
IDDM: insulin-dependent diabetes
LBBB: left bundle branch block
MI: myocardial infarction
NRI: Net Reclassification Improvement
NSQIP: National Surgical Quality Improvement Program
OR: Odds Ratio
RCRI: the Revised Cardiac Risk Index
ROC: Receiver Operating Characteristic
TIA: transient ischemic attack
URL: upper reference limit
