# Supplementary material for: Combining CHA2DS2-VASc score into RCRI for prediction perioperative cardiovascular outcomes in patients undergoing non-cardiac surgery: a retrospective pilot study
Source: BMC Anesthesiol. 2021 Nov 9;21:276. doi: 10.1186/s12871-021-01496-2 (PMC8576929; doi:10.1186/s12871-021-01496-2)
Supplement: Supplementary file 1 — Additional file 1. [file 12871_2021_1496_MOESM1_ESM.docx]

**Combining CHA_2_DS_2_-VASc score into RCRI for prediction perioperative cardiovascular outcomes in patients undergoing non-cardiac surgery:** **a retrospective pilot study**

Song-Yun Chu, MD, Pei-Wen Li, MD, Fang-Fang Fan, MD, Xiao-Ning Han, MD, Lin Liu, MD, Jie Wang, MD, Jing Zhao, MD, Xiao-Jin Ye, MD, Wen-Hui Ding, MD

**Supplementary Table 1. Association of the clinical characteristics with perioperative cardiovascular outcomes in patients undergoing intra-abdominal surgery**

|  | **MI** | | | | **HF** | | | | **AF** | | | | **Stroke*** | |
| --- | --- | --- | --- | --- | --- | --- | --- | --- | --- | --- | --- | --- | --- | --- |
|  | **Unadjusted**  **OR (95%CI)** | **P value** | **Adjusted**  **OR (95%CI)** | **P value** | **Unadjusted**  **OR (95%CI)** | **P value** | **Adjusted**  **OR (95%CI)** | **P value** | **Unadjusted**  **OR (95%CI)** | **P value** | **Adjusted**  **OR (95%CI)** | **P value** | **Unadjusted**  **OR (95%CI)** | **P value** |
| **RCRI scheme** |  |  |  |  |  |  |  |  |  |  |  |  |  |  |
| **Scr >2mgl/dL** | 9.562(2.936-31.140) | <0.001* | 5.922(1.643-21.354) | 0.007* | 9.134(1.916-43.542) | 0.006* | 6.208(1.210-31.838) | 0.029* | 3.579(0.450-28.490) | 0.228 | 1.680(0.185-15.292) | 0.645 | - | - |
| **IDDM** | 2.079(0.614-7.037) | 0.239 | 0.687(0.173-2.722) | 0.593 | 3.191(0.709-14.350) | 0.130 | 2.076(0.426-10.108) | 0.366 | 1.307(0.171-10.000) | 0.797 | 0.292(0.028-3.050) | 0.304 | - | - |
| **CHF** | 10.384(3.154-34.189) | <0.001* | 4.663(1.139-19.093) | 0.032* | 4.399(0.547-35.375) | 0.164 | 3.660(0.410-32.664) | 0.245 | 8.940(1.879-42.541) | 0.006* | 7.869(1.118-55.399) | 0.038* | - | - |
| **CHD** | 5.609(2.829-11.121) | <0.001* | 2.721(1.230-6.019) | 0.013* | 11.880(0.535-6.605) | 0.325 | 0.842(0.219-3.234) | 0.802 | 2.371(0.778-7.229) | 0.129 | 0.963(0.274-3.380) | 0.953 | 3.112(0.321-30.190) | 0.327 |
| **Cerebrovascular disease** | 2.712(1.020-7.208) | 0.046* | 1.661(0.584-4.729) | 0.342 | 3.730(1.048-13.274) | 0.042* | 2.594(0.689-9.772) | 0.159 | 4.745(1.533-14.689) | 0.007* | 4.208(1.240-14.278) | 0.021* | 18.545(2.564-134.139) | 0.004* |
| **CHA_2_DS_2_-VASc Score scheme** |  |  |  |  |  |  |  |  |  |  |  |  |  |  |
| **Age≥65** | 4.677(2.262-9.671) | <0.001* | 2.563(1.112-5.905) | 0.027* | 3.005(1.119-8.067) | 0.029* | 1.912(0.650-5.620) | 0.239 | 8.656(2.521-29.718) | 0.001* | 5.263(1.419-19.518) | 0.013* | 4.448(0.461-42.902) | 0.197 |
| **Age≥75** | 6.982(3.662-13.312) | <0.001* | 3.590(1.729-7.452) | 0.001* | 5.890(2.302-15.071) | <0.001* | 4.647(1.691-12.768) | 0.003* | 7.293(2.972-17.894) | <0.001* | 4.394(1.548-12.472) | 0.005* | 5.677(0.794-40.591) | 0.084 |
| **Female gender** | 0.893(0.469-1.702) | 0.732 | 0.873(0.423-1.801) | 0.713 | 1.697(0.665-4.335) | 0.269 | 1.736(0.663-4.547) | 0.262 | 2.046(0.829-5.046) | 0.120 | 2.826(1.028-7.767) | 0.044 | 4.057(0.421-39.127) | 0.226 |
| **Hypertension** | 2.828(1.498-5.336) | 0.001* | 1.123(0.529-2.382) | 0.763 | 2.490(0.979-6.333) | 0.055 | 1.349(0.479-3.801) | 0.571 | 1.325(0.524-3.353) | 0.552 | 0.377(0.130-1.094) | 0.073 | - | - |
| **CHF** | 10.384(3.154-34.189) | <0.001* | 4.663(1.139-19.093) | 0.032* | 4.399(0.547-35.375) | 0.164 | 3.660(0.410-32.664) | 0.245 | 8.940(1.879-42.541) | 0.006* | 7.869(1.118-55.399) | 0.038* | - | - |
| **DM** | 2.072(0.991-4.329) | 0.053 | 0.911(0.398-2.088) | 0.826 | 3.084(1.140-8.345) | 0.027 | 2.045(0.709-5.899) | 0.186 | 3.341(1.311-8.511) | 0.011* | 1.658(0.583-4.718) | 0.344 | 6.072(0.849-43.432) | 0.072 |
| **Ischemic stroke/TIA** | 3.015(1.130-8.047) | 0.028* | 1.907(0.662-5.495) | 0.232 | 2.527(0.566-11.295) | 0.225 | 1.677(0.357-7.879) | 0.513 | 5.266(1.695-16.354) | 0.004* | 5.123(1.471-17.848) | 0.010* | 20.50(2.829-148.538) | 0.003* |
| **Vascular disease** | 5.229(2.643-10.348) | <0.001* | 2.237(1.036-4.832) | 0.040* | 2.547(0.824-7.878) | 0.105 | 1.074(0.314-3.678) | 0.909 | 2.224(0.730-6.775) | 0.159 | 0.713(0.196-2.596) | 0.608 | 2.924(0.302-28.354) | 0.355 |

**Abbreviations: MI: myocardial infarction; HF: heart failure; AF: atrial fibrillation; CHD: coronary heart disease; CHF: congestive heart failure; DM: diabetes; IDDM: insulin-dependent diabetes; TIA: transient ischemic attack; Scr: serum creatinine; OR: odds ratio; CI: confidence interval.**

(Continued)

|  | **Total cardiac ischemic events** | | | | **Composite cardiovascular events** | | | | **All-cause death** | | | |
| --- | --- | --- | --- | --- | --- | --- | --- | --- | --- | --- | --- | --- |
|  | **Unadjusted**  **OR(95%CI)** | **P value** | **Adjusted**  **OR(95%CI)** | **P value** | **Unadjusted**  **OR(95%CI)** | **P value** | **Adjusted**  **OR(95%CI)** | **P value** | **Unadjusted**  **OR(95%CI)** | **P value** | **Adjusted**  **OR(95%CI)** | **P value** |
| **RCRI scheme** |  |  |  |  |  |  |  |  |  |  |  |  |
| **SCr >2mg/dL** | 7.035(2.186-22.641) | 0.001* | 5.035(1.390-18.244) | 0.014* | 5.679(1.924-16.761) | 0.002* | 3.126(0.946-10.328) | 0.062 | 12.987(3.925-42.976) | <0.001* | 8.441(2.152-33.111) | 0.002* |
| **IDDM** | 2.169(0.744-6.324) | 0.156 | 0.776(0.221-2.727) | 0.692 | 2.529(1.087-5.884) | 0.031* | 0.997 (0.367-2.711) | 0.996 | 1.802(0.415-7.828) | 0.432 | 1.263(0.255-6.254) | 0.775 |
| **CHF** | 10.819(3.556-32.916) | <0.001* | 4.593(1.245-16.948) | 0.022* | 11.375(4.017-32.210) | <0.001* | 7.040(2.005-24.716) | 0.002* | 5.692(1.226-26.428) | 0.026* | 2.889(0.440-18.976) | 0.269 |
| **CHD** | 6.926(3.797-12.635) | <0.001* | 3.707(1.836-7.483) | <0.001* | 4.595(2.715-7.776) | <0.001* | 1.953(1.051-3.628) | 0.034* | 1.444(0.494-4.220) | 0.502 | 0.708(0.209-2.402) | 0.580 |
| **Cerebrovascular disease** | 3.629(1.621-8.126) | 0.002* | 2.274(0.945-5.471) | 0.067 | 4.497(2.346-8.620) | <0.001* | 3.050(1.494-6.225) | 0.002* | 4.892(1.915-12.496) | 0.001* | 3.811(1.385-10.490) | 0.010* |
| **CHA_2_DS_2_-VASc Score scheme** | |  |  |  |  |  |  |  |  |  |  |  |
| **Age≥65** | 3.882(2.103-7.166) | <0.001* | 2.100(1.029-4.288) | 0.014* | 4.036(2.469-6.598) | <0.001* | 1.991(1.114-3.557) | 0.020* | 2.264(1.079-4.750) | 0.031* | 1.143(0.488-2.676) | 0.759 |
| **Age≥75** | 5.965(3.364-10.577) | <0.001* | 3.483(1.761-6.891) | <0.001* | 6.233(3.895-9.976) | <0.001* | 3.401(1.950-5.932) | <0.001* | 3.965(1.872-8.397) | <0.001* | 1.911(0.809-4.513) | 0.139 |
| **Female gender** | 1.071(0.611-1.877) | 0.811 | 1.095(0.580-2.066) | 0.780 | 1.344(0.859-2.105) | 0.196 | 1.513(0.905-2.530) | 0.114 | 0.894(0.426-1.876) | 0.768 | 0.940(0.421-2.102) | 0.881 |
| **Hypertension** | 3.037(1.731-5.329) | <0.001* | 1.272(0.658-2.457) | 0.474 | 2.891(1.838-4.545) | <0.001* | 1.177(0.685-2.024) | 0.555 | 2.195(1.058-4.553) | 0.035* | 1.665(0.722-3.839) | 0.232 |
| **CHF** | 10.819(3.556-32.916) | <0.001* | 4.593(1.245-16.948) | 0.022* | 11.375(4.017-32.210) | <0.001* | 7.040(2.005-24.716) | 0.002* | 5.692(1.226-26.428) | 0.026* | 2.889(0.440-18.976) | 0.269 |
| **DM** | 2.334(1.233-4.419) | 0.009* | 1.056(0.514-2.169) | 0.881 | 2.897(1.750-4.796) | <0.001* | 1.426(0.799-2.545) | 0.229 | 0.922(0.317-2.679) | 0.881 | 0.515(0.164-1.617) | 0.256 |
| **Ischemic stroke/TIA** | 3.395(1.450-7.946) | 0.005* | 1.970(0.756-5.131) | 0.165 | 4.557(2.325-8.932) | <0.001* | 3.009(1.399-6.469) | 0.005* | 4.264(1.563-11.634) | 0.005* | 2.638(0.832-8.361) | 0.099 |
| **Vascular disease** | 7.053(3.891-12.782) | <0.001* | 3.103(1.590-6.058) | 0.001* | 4.561(2.716-7.660) | <0.001* | 1.745(0.966-3.154) | 0.065 | 1.779(0.667-4.745) | 0.250 | 0.806(0.267-2.435) | 0.702 |

*There were only 4 cases of perioperative stroke events. All the patients had a hypertension history. Neither of them had a history of heart failure, insulin-dependent diabetes or elevated serum creatinine. No further multivariate analysis was attempted.

**Supplementary Table 2. Perioperative cardiovascular events rates in the study population stratified by RCRI**

| **Endpoints** | **Overall** |  | **RCRI ^a^** | | | **P for trend** |
| --- | --- | --- | --- | --- | --- | --- |
|  |  | **0** | **1** | **2** | **≥3** |  |
| **Patients No (%)** | 1079 | 0(0) | 889(82.4) | 152(14.1) | 38(3.5) |  |
| **Myocardial infarction** | 40(3.7) | 0(0) | 20(2.2) | 12(7.9) | 8(21.1) | <0.001 |
| **Total ischemic events** | 52(4.8) | 0(0) | 24(2.7) | 18(11.8) | 10(26.3) | <0.001 |
| **Heart failure** | 18(1.7) | 0(0) | 9(1.0) | 7(4.6) | 2(5.3) | 0.001 |
| **Atrial fibrillation** | 20(1.9) | 0(0) | 13(1.5) | 2(1.3) | 5(13.2) | 0.001 |
| **Ischemic stroke** | 4(0.4) | 0(0) | 2(0.2) | 1(0.7) | 1(2.6) | 0.047 |
| **All-cause death** | 30(2.8) | 0(0) | 18(2.0) | 7(4.6) | 5(13.2) | <0.001 |
| **Composite endpoints ^b^** | 83(7.7) | 0(0) | 44(4.9) | 24(15.8) | 15(39.5) | <0.001 |

a. RCRI: Revised Cardiac Risk Index, includes renal insufficiency (creatinine≥2 mg/dL), Insulin-dependent diabetes mellitus, heart failure, ischemic heart disease, cerebrovascular accident or TIA, intra-thoracic, intra-abdominal, or supra-inguinal vascular surgery; each one calculated as 1 score.

b. Composite end point of total ischemic events, heart failure, arrhythmia, ischemic stroke, transient ischemic attack or systemic embolism, and death.

**Supplementary Table 3. Perioperative cardiovascular events rates in the study population stratified by CHA_2_DS_2_-VASc Score**

| **Endpoints** | **Overall** |  | **CHA_2_DS_2_-VASc Score ^a^** | | | **P for trend** |
| --- | --- | --- | --- | --- | --- | --- |
|  |  | **0** | **1** | **2** | **≥3** |  |
| **Patients No (%)** | 1079 | 288(26.7) | 348(32.3) | 210(19.5) | 233(21.6) |  |
| **Myocardial infarction** | 40(3.7) | 1(0.3) | 7(2.0) | 9(4.3) | 23(9.9) | <0.001 |
| **Total ischemic events** | 52(4.8) | 2(0.7) | 8(2.3) | 11(5.2) | 31(13.3) | <0.001 |
| **Heart failure** | 18(1.7) | 2(0.7) | 3(0.9) | 4(1.9) | 9(3.9) | 0.005 |
| **Atrial fibrillation** | 20(1.9) | 1(0.3) | 2(0.6) | 6(2.9) | 11(4.7) | <0.001 |
| **Ischemic stroke** | 4(0.4) | 0(0) | 0(0) | 1(0.5) | 3(1.3) | 0.042 |
| **All-cause death** | 30(2.8) | 3(1.0) | 7(2.0) | 7(3.3) | 13(5.6) | 0.002 |
| **Composite endpoints ^b^** | 83(7.7) | 6(2.1) | 12(3.4) | 18(8.6) | 47(20.2) | <0.001 |

a CHA_2_DS_2_-VASc score is calculated as congestive heart failure (1 point), hypertension (1 point), age 75 years or older (2 points), diabetes (1 point), stroke/transient ischemic attack/thromboembolism (2 points), vascular disease (prior myocardial infarction, peripheral artery disease, or aortic plaque; 1 point), age 65 to 75 years (1 point), female sex (1 point).

b Composite end point of total ischemic events, heart failure, arrhythmia, ischemic stroke, transient ischemic attack or systemic embolism, and death.
